# Community partner coauthorship for increased implementation science impact: Strengthening capacity and engagement

**DOI:** 10.1017/cts.2024.574

**Published:** 2024-09-20

**Authors:** Rebekka M. Lee, Cristina Huebner Torres, James G. Daly, Annette Thomas, Peggy A. Hannon, Sonja Likumahuwa-Ackman, Karen M. Emmons

**Affiliations:** 1 Harvard T.H. Chan School of Public Health, Boston, MA, USA; 2 Caring Health Center, Springfield, MA, USA; 3 Brockton Neighborhood Health Center, Brockton, MA, USA; 4 University of Washington, Seattle, WA, USA; 5 Oregon Health & Science University, Portland, OR, USA

**Keywords:** Implementation science, partnership, community engagement, capacity building, coauthorship, dissemination

## Abstract

Engaging diverse partners in each phase of the research process is the gold standard of community-engaged research and adds value to the impact of implementation science. However, partner engagement in dissemination, particularly meaningful involvement in developing peer-reviewed manuscripts, is lacking. The Implementation Science Centers in Cancer Control are using the Translational Science Benefits Model to demonstrate the impact of our work beyond traditional metrics, including building capacity and promoting community engagement. This paper presents a case example of one center that has developed a policy for including community partners as coauthors. Standard practices are used to foster clear communications and bidirectional collaboration. Of published papers focused on center infrastructure and implementation research pilots, 92% have community partner coauthors. This includes 21 individuals in roles ranging from physician assistant to medical director to quality manager. Through this intentional experience of co-creation, community partners have strengthened implementation science expertise. Community coauthors have also ensured that data interpretation and dissemination reflect real-world practice environments and offer sustainable strategies for rapid translation to practice improvements. Funders, academic journals, and researchers all have important roles to play in supporting community coauthors as critical thought partners who can help to narrow the gap between research and practice.

## Introduction

Engaging diverse partners in each phase of the research process is the gold standard of community-engaged research and has been shown to increase the relevance and impact of implementation science [1]. However, a recent review of community partner engagement in different phases of research found that partners were least likely to be engaged in the dissemination of research findings – only 30% of partnered studies engaged partners in dissemination, while almost half of partnered studies engaged partners in identifying research questions and developing study protocols [2]. The field of implementation science has a robust and growing body of literature describing practical approaches to meaningful partner engagement throughout the research phases, with a particular focus on the importance of engagement in understanding context, selecting implementation strategies, and monitoring implementation success [3–5]. Structured approaches to partner engagement in the dissemination phase, particularly processes to encourage and ensure meaningful involvement in developing peer-reviewed manuscripts, are lacking. This lack of structured approaches for including community authors in dissemination raises the risk that community partners are inadvertently left out of the dissemination process or included in a haphazard way that limits their contributions. It also reduces the likelihood that study findings are interpreted in ways that reflect the on-the-ground implementation team’s perspectives. This ultimately means that community partners’ expertise to effect change in practice settings is not adequately or accurately represented in the literature.

The Translational Science Benefits Model (TSBM) was developed at the Institute of Clinical and Translational Sciences at the School of Medicine and the Brown School at Washington University in St Louis, Missouri, United States. It is a framework of 30 indicators across four domains (clinical, community, economic, and policy) that are designed to measure the impact of public health and clinical research in a variety of settings [6]. The Implementation Science Centers in Cancer Control (ISC^3^) have adapted the TSBM to demonstrate the impact of our work beyond traditional research metrics by adding a new outcomes domain that captures implementation science disciplinary impact. Implementation science outcomes serve as a precursor to the model’s established domains of impact. Including these outcomes will help to sharpen the focus on the translational steps needed to achieve a broad range of impacts. Two such implementation outcomes are building capacity and promoting community engagement.

Academic institutions and community partners that are part of ISC^3^ bring decades of experience conducting research that centers the community, ensuring that research questions emerge from real-world challenges and findings are translated into health care programs, services, and quality improvements [7–11]. This has been achieved by including community coinvestigators (e.g., patients, clinical providers, and staff in community-based organizations) in research studies, embedding research leadership and staff into health centers, integrating quality improvement and implementation science, creating structured groups of people with lived experience for engagement throughout the research process, developing training for research teams and patients to level power, and crafting fair and transparent compensation structures that center the needs and preferences of patient advisors [7–11].

One ISC^3^ Center has operationalized these new implementation outcomes to TSBM, building capacity and promoting community engagement, by accelerating community partner engagement in dissemination through inclusive authorship practices and policies [11]. These practices and policies provide transparency, accountability, and assurance that community partner values are centered in our research. This paper presents a case example of how this center has engaged implementation partners, such as health center leaders, quality improvement staff, and community health workers, as coauthors. We will describe the methods and results from this approach and then highlight key lessons learned for the implementation science field. We hope that sharing this successful process helps more implementation scientists adopt this process or develop their own to increase the engagement of their community partners in disseminating findings and maximize the potential impact of their work.

## Materials and methods

### Case example

**Figure 1.**
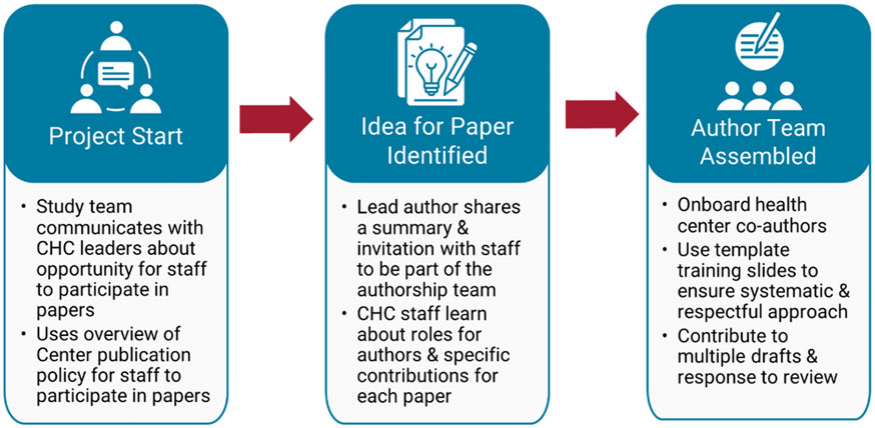
Step-by-step process for Implementation Science Center for Cancer Control Equity community partner coauthorship.

A replicable step-by-step process has been developed to ensure consistent, collaborative, and respectful coauthorship at the Implementation Science Center for Cancer Control Equity (ISCCCE) (see Fig. 1). The center is grounded in a robust partnership with the Massachusetts League of Community Health Centers, the primary care association for the state, and aims to increase the use of evidence-based strategies for cancer prevention and control in community health centers. Community health centers are also core partners, forming an Implementation Learning Community, and partnering on or leading a series of pilot research projects over the five-year grant period. Center leaders in the administrative core have developed a coauthorship policy that applies to all papers describing implementation research pilots and center infrastructure. Commentaries and ancillary papers written with minimal center funding are not included. At the initiation of each pilot project, the study team uses a partner-facing overview of the publication policy (Supplementary Materials) to communicate with organizational leaders about the intention of providing the opportunity for staff to participate in papers.

Once an idea for a manuscript has been identified, the lead author shares a summary and invitation with health center partners to join the authorship team. Partners, ranging in experience and expertise with dissemination, learn about typical roles for manuscript authors and the specific contributions for each paper via a concrete bulleted list of ways in which community partners can participate on the coauthorship team that is tailored to the paper topic and type (Supplementary Materials). For implementation studies, the lead author (who could be an external or internal to the health center) invites health center staff who were part of the research project as coauthors. All health center staff who make a substantial contribution to the data collection and implementation of the project are encouraged to participate. On conceptual, methodological, or observational studies in which health center staff are less actively involved in the daily research activities, the lead author puts out a broader call for community partner coauthors to health center practitioners interested in the topic with outreach support from the Massachusetts League of Community Health Centers staff.

Once coauthors are identified, the lead author hosts an introductory meeting using template slides to ensure a systematic and respectful onboarding process and provides support to partners who are new to the paper writing process (Supplementary Materials). These slides include aims and data collected, paper timeline and expectations, and roles of all coauthors. Those who are new to academic paper writing also benefit from an overview of the general writing, submission, and revision process. The key messages of the introductory training are also reiterated in an email with clear, tailored guidance on community coauthor contributions (Supplementary Materials). In addition to peer-reviewed manuscripts, we produce lay summaries of our publications using compelling visuals, guided by a plain language checklist [12] to ensure accessibility and further support actionable dissemination of findings [13]. These summaries have been shared at the annual meeting of Massachusetts health centers and via one-on-one meetings with health center teams.

In addition to this center-wide approach, several exemplar best practices have emerged as part of some projects. For example, the ISCCCE approach to community partner coauthorship has presented the capacity-building opportunity for teams to improve their paper writing skills together. In a paper assessing clinical-community linkages for evidence-based cancer prevention, health center staff across a range of roles (e.g., a public health and policy leader, quality improvement manager, and social epidemiologist/administrator) were invited to collaborate as coauthors. A team-based approach among the community partners was used to engage in the manuscript review, contributions of health center-specific context, and offering additional resources to frame the manuscript findings and implications for translation [14].

A second exemplar best practice for partner engagement developed through ISCCCE has been drafting data use agreements that emphasize community partner priorities and voice in their development. One example is outlined in Table 1 in which ISCCCE and Caring Health Center revised the legal language of the data use agreement to reflect equitable research partnership and dissemination priorities. The Health Center’s research leadership and their legal counsel drafted revised language illustrative of the health center as a research partner. The ISCCCE research leadership worked with their legal office and advocated within the academic setting in support of the revisions. Together, the team created a revised data use agreement with language that more accurately represented the health center, its role, and its underlying mission-based approach to research.

**Table 1.** Data use agreement (DUA) changes for community-academic research

Standard Academic DUA Legalese	FQHC-Academic Partnership Revised DUA Legalese
FQHC = “Data Provider”	FQHC = “Research Partner”
Definition of “researcher” included for academic partner only	Added definition of “community researcher”
Ownership of data/dissemination process	Collaborators in analytics and dissemination process (data owned by the patients)
Statement that dissemination could not be withheld based on findings (e.g., intellectual freedom)	Statement revised to ensure dissemination strategy to consider possible unintended consequences

## Results

Of published papers focused on center infrastructure and implementation research pilots, 92% (12 of 13 papers) have community partner coauthors. Papers have been published in a broad range of high-impact journals with open-access policies to support practice-based dissemination. This includes 21 individuals in job roles ranging from physician assistant to medical director to quality manager (Table 2). Nine individuals (43%) have been engaged in multiple ISCCCE papers.

**Table 2.** Job roles of Implementation Science Center for Cancer Control Equity community partner coauthors

Job role	Example title	N
*Community health center*		
Quality improvement	Quality and Performance Improvement Manager	4
Leadership	Deputy CEO	3
Provider	Physician Assistant	2
Community health worker	Prevention Specialist	2
Policy & advocacy	Director of Public Health Practice, Policy, and Advocacy	1
*Primary care association*		
Health informatics	Community Health Data Manager	4
Leadership	Chief Operating Officer	3
*Primary care practice*		
Leadership	Medical Director	1
*Community-based organization*		
Leadership	Executive Director	1

**Figure 2.**
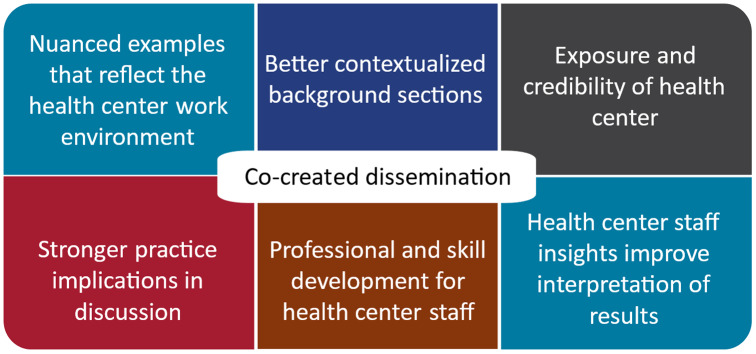
Benefit of community partner-researcher coauthorship reported by Implementation Science Center for Cancer Control Equity investigators and partners gathered via an Implementation Learning Community meeting and the writing team.

ISCCCE researchers and community partners have described a range of benefits gained through the center’s approach to coauthorship (Fig. 2). Through this intentional experience of co-creation, health center staff gain implementation science expertise and enhanced contextualization of their work – being able to see how their work fits into the bigger picture of the state of the science. The community health centers also benefit – coauthorship improves the exposure and credibility of their organization, which can be showcased in branding and on websites to attract talented clinical staff. Simultaneously, papers have been improved with better contextualized background sections, more nuanced examples that reflect the real, dynamic, and complex work environment of community health centers, improved interpretation of results and accuracy from the community partner perspective, and stronger practice implications as part of the discussion. With these improvements to papers, come education benefits for the research team that can be applied to current and future implementation science projects.

## Discussion

The policies and practices described highlight the value of engaging implementation science practice partners in the co-creation of dissemination products. Including practice partners as critical thought partners has the potential of narrowing the gap between research and practice. Community partner coauthorship demonstrates the value of the partnership at the center of the research and acknowledges research being conducted in practice settings like community health centers. Potential benefits to staff include formal recognition for the research collaboration, learning about the publication process, expanded professional skills, and increased opportunity for advancement. Capacity building extends beyond individual staff, to strengthen teams and health centers through collaborative coauthorship opportunities. Having community coauthors ensures that interpretations of findings reflect practitioner real-world experiences. It centers what is most important to community health centers (i.e., patient safety, quality of care) in publications and the collaborative experience helps partners to design high quality initiatives in their own practice. Partner coauthorship may also facilitate dissemination of research findings into communities. While many community-engaged researchers may see the value of including partners as coauthors and may do so, we find it useful to formalize this process. This case study illustrates a replicable approach that could encourage more researchers to collaborate with community partners in research dissemination. Researchers already including community partners in research dissemination could adopt or adapt our tools to do so in a more systematic and inclusive way.

There are a range of factors that may present barriers to effective community partner-researcher coauthorship, and therefore, should be considered from the onset of a community-engaged research project. One barrier for both researchers and partners is the perceived time commitment to collaborate on manuscript development. Communicating clear expectations for coauthorship, including time commitments, and aligning specific contributions with expertise, interest, and bandwidth when assembling the author team can help make for a more efficient process and allow for broader participation. Creating coauthor roles that fit with partners’ expertise, rather than requiring a standard approach across all people and papers, will likely avoid wasted time and frustration. Another challenge is that some partner organizations may have specific administrative processes in place that must be completed before staff can be included as authors or to determine if partner organizations can be named in dissemination. By asking about any organizational policies or standard practices around authorship early on, researchers can factor this into submission timeline. There can also be barriers related to the journals selected for publication. To ensure community partners have access to the manuscript once published, researchers should budget for fees that often accompany open access journal in grant proposals. Lastly, researchers should consider the maximum number of authors when selecting a journal for submission to help inform the size of their author team. ISCCCE has also included “The ISCCCE Consortium” or “for the ISCCCE Partners(hip)” as a listed author in manuscripts to acknowledge those who did not participate as coauthors but contributed to the research. This enables collaborators to cite publications on their resumes if they are interested.

This paper presents the initial application of the ISCCCE formal partnered coauthorship process. We recognize that many of the community partner coauthors to date have been in leadership positions and we intend to develop strategies for engaging more patient-facing staff such as community health workers, nurses, and medical assistants. In the future, we plan to conduct a robust evaluation of the approach beyond counting the number of people and publications impacted, to include the perceived benefits to each community partner engaged in dissemination. This evaluation would take a mixed methods approach – combining demographics and role of coauthors and ratings of engagement levels from validated survey measures with open-ended questions describing the coauthorship experience. We also intend to extend and adapt this model beyond implementation research with health center partners to include individuals in non-clinical community setting who may have different barriers to participation such a less formal education or different competing demands.

The full benefits of community partner coauthorship will not be met without intentional participation across the scientific community, including funders, journals, and academic institutions. While some funders collect data on community coauthorship, it is unclear whether and how these data are used; other funders do not measure community coauthorship. Funders can and should provide clear and meaningful incentives for community collaboration in dissemination; for example, researchers’ track record and meaningful plans for community collaboration in dissemination could be included in review criteria for grants featuring community and clinical partnerships. Furthermore, academic journals have a role to play in ensuring community partners are valued as coauthors. Their author guidelines should create a more inclusive approach to what it means to contribute to generalizable knowledge (e.g., contributions to interpretation in team meetings, implications to workflows, good fit adaptations to study protocols) and remove barriers and create experience-based credentials for community coauthors that correspond to commonly expected credentials such as author degree requirements and limits on the number of coauthors. Academic institutions should also recognize researchers’ collaboration with partners, including equitable coauthorship, as part of the promotion and tenure process and/or annual metric review process. For example, academic institutions could have faculty describe these efforts as part of their overall efforts to improve equity through community partnerships. Finally, researchers create space for authentic collaboration when they approach coauthorship with curiosity and humility. Implementation scientists should engage with community partners as critical thought partners in alignment with the field’s goal of narrowing the gap between research and practice advancements.

## Supporting information

Lee et al. supplementary material Lee et al. supplementary material

## Supplementary material

The supplementary material for this article can be found at https://doi.org/10.1017/cts.2024.574.
