# Supplementary material for: Community partner coauthorship for increased implementation science impact: Strengthening capacity and engagement
Source: J Clin Transl Sci. 2024 Sep 20;8(1):e129. doi: 10.1017/cts.2024.574 (PMC11428058; doi:10.1017/cts.2024.574)
Supplement: Lee et al. supplementary material [file S2059866124005740sup001.pdf]

Publication Policy Overview—Involvement of Community Partners

The Implementation Science Center for Cancer Control Equity (ISCCCE) and Rapid Acceleration of COVID Diagnostics in Massachusetts (RADX-MA) are initiatives led by Dr. Karen Emmons at the Harvard T.H. Chan School of Public Health, Dr. Elsie Taveras at the Kraft Center for Community Health at Massachusetts General Hospital, and Susan Dargon-Hart at the Massachusetts League of Community Health Centers. Broadly, the goals of ISCCCE are to develop and test strategies to improve cancer prevention and control practices and health equity in Massachusetts community health centers. The goal of RADX-MA is to implement a community-partnered COVID-testing strategy to increase the number of tests completed.

We have developed approaches to help ensure equitable participation in study products, including guidelines for including community partners on author teams. For both of these initiatives, papers will include "for the ISCCCE (or RADx-MA) Partnership" in the authorship line so that all CHC staff that have been involved with the project may include the papers on their resumes if they wish. In addition, we anticipate that in most cases one or two CHC staff members may participate as named authors on a given paper, to the extent that there is interest.

Once a publication idea has been identified, the lead author will share a summary of the idea with the CHC project leads, via the I-Lab, and ask them to determine if any CHC staff wish to be part of the authorship team. We will in particular ensure inclusion of those staff members that have identified a particular interest in the topic. It is the lead author's responsibility to ensure that CHC staff members are invited into the manuscript development process in a timely, respectful and authentic manner.

Typical roles for manuscript authors may include any of the following:

- Ongoing participation in authorship team communication
- Contributions to the interpretation of study findings
- Informing the framing of implementation or findings in local or broader context
- Participation in drafting of manuscripts sections, such as background, methodology, results, or discussion/conclusion
- Reviewing the manuscript for accuracy and relevance

Sample community co-author outreach emails

Initial outreach

Dear [partner name],

I'm writing to let you know about an opportunity to participate on the authorship team for a forthcoming [research project] paper. The proposed paper title, lead author, and research questions are outlined below. In the attached Publication Policy Overview, you will find guidelines for involvement of community partners in developing study products, including typical roles for participation on the authorship team.

Please let me know if you are interested in being a named author and participating on the authorship team by [date]. Regardless of whether or not you wish to be an author, papers will include "For the [research project] Partnership" in the authorship line so that all partners that have been involved with the project will have the opportunity to include papers on resumes and CVs.

Proposed Paper Title: Addressing COVID-19 testing inequities among underserved populations in "hotspot" communities across Massachusetts: a qualitative exploration of health center staff, partner, and resident perceptions

Lead Author: Rebekka Lee

Proposed Research Questions:

(1) What are the perceptions of COVID-19 testing barriers among community health center staff, partners, and residents?

(2) How can community-identified needs and assets be translated to build tailored clinical-community strategies for addressing COVID-19 testing inequities?

Thank you for considering and for being such valuable thought partners for our [research project/center]!

Best,

[Lead author]

Follow up email after intro meeting

Thank you all for a great meeting yesterday!

Attached you'll find the latest draft of our paper, along with 2 PDF figures detailing our process and overall results, and an example of one of the tables summarizing the results.

Areas I'd love feedback on are:

- Examples to include in our data for action section:
  - What specific ways could highlight how you used the data from the qualitative summaries provided to implement your tailored approach? For example, today [partner] mentioned how the qualitative interviews helped them narrow down the members of their local CAB.
  - Are there findings specific to your health center that you found most useful for action in your community? Could you summarize in 2-3 sentences?
- Presentation of the data in the table example and figure 2:
  - Does this seem like a good way for us to show the heaps of data we've collected?
  - How could we improve these visualizations to be more digestible?
- Additional information about how health centers are contributing to the COVID-19 response for the introduction.
- General framing of the paper.
- Points you want to ensure are included in the discussion.

Please use comments and track changes to add your thoughts by [2 weeks to 1 month from this email].

All the best,

[Lead author]

Example partner co-author introductory meeting slides

Adapt this slide template for an initial 30-minute meeting with partner co-authors

Use the notes as a guide for what to present and discuss

ISCCCE
IMPLEMENTATION SCIENCE CENTER FOR CANCER CONTROL EQUITY

Addressing COVID-19 testing inequities among underserved populations in "hotspot" communities across Massachusetts: A qualitative exploration of health center staff, partner, and resident perceptions

October 21, 2021

Rebekka Lee, ScD

ISCCCE
IMPLEMENTATION SCIENCE CENTER FOR CANCER CONTROL EQUITY

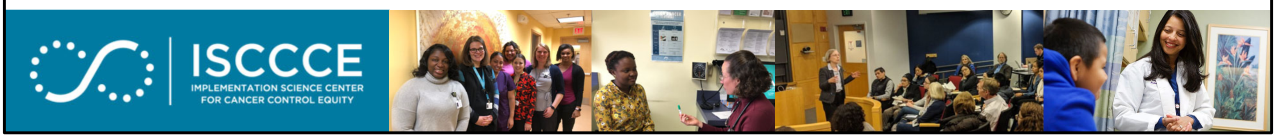

Intros – 5 minutes **make sure to allow time for all co-authors to meet and introduce themselves before jumping into the content**
Paper status – 10 minutes (slides 2-4)
CHC author contribution review – 5 minutes (slide 5)
Action items and questions – 10 minutes (slide 6/7)

Paper aims

- Explore the perceptions of COVID-19 testing barriers among community health center staff, community partners, and residents gathered through a rapid needs assessment.
- Describe how these community-identified needs and assets can be translated to build tailored clinical-community strategies for addressing testing inequities.

ISCCCE
IMPLEMENTATION SCIENCE CENTER FOR CANCER CONTROL EQUITY

Walk through the aims for the paper with the team
Pause to answer any clarifying questions

Paper overview

- 107 community health center staff (N=12), partners (N=57) and residents (N=38) participated in the project across 84 interviews
- Interviews conducted in English, Spanish, Vietnamese, and Arabic
- Present a 2-phase approach
  - Data for action – aggregate reports for implementation & communications teams, site-specific summaries & presentations for each community
  - In depth thematic analysis – generalized findings about COVID-19 testing barriers and solutions organized by the social ecological model

ISCCCE
IMPLEMENTATION SCIENCE CENTER FOR CANCER CONTROL EQUITY

4

Review methods and analysis
Include who data was collected from (e.g., staff, partners, residents, patients), type of data (e.g., EMR, surveys, interviews, observations), and basic overview of analysis

Paper status

- ✓ Paper proposal approved
- ✓ Invite author team
- ✓ Select target journal
- ✓ Draft introduction
- ✓ Draft methods
- ✓ Conduct analysis
- ✓ Develop tables & figures

- ❑ Review results interpretation with author team
- ❑ Draft results
- ❑ Draft discussion
- ❑ Paper edits from author team

Target for submission:
August 1

ISCCCE
IMPLEMENTATION SCIENCE CENTER FOR CANCER CONTROL EQUITY

5

Include this list of paper milestones (ok to add new milestones specific for your paper)
Change checkmarks and open boxes to indicate the current paper status
Update target date for submission

Community partner authorship

- Prioritized within Publication Policy of ISCCCE & RADx-MA
- Your role
  - Ongoing participation in authorship team communication
  - Contributions to the interpretation of study findings
  - Informing the framing of findings in local or broader context
  - Participation in drafting of manuscripts sections, such as **background,** methodology, **results, or discussion/conclusion**
  - Reviewing the manuscript for accuracy and relevance

ISCCCE IMPLEMENTATION SCIENCE CENTER FOR CANCER CONTROL EQUITY

6

Review Center publication policy and role of partner co-authors
Bold/underline key role for authors on this specific paper

In this example, CHC partners improved the description of CHC role in the response to the COVID-19 pandemic in the introduction, drafted examples of how data was used for action in the results, and contextualized the thematic analysis findings on barriers and solutions to their real-world practice.

Before moving to next steps, open up discussion – do partner co-authors have other ideas of how they could contribute to the paper?

Next steps

- Bekka circulate latest draft for team review: TOMORROW
- CHC co-authors confirm interest in participation: July 16
- Writing team provide feedback: July 23
  - Accuracy & relevance
  - Interpretations of results
  - Ideas of points to highlight in the discussion
  - CHC co-authors provide input on how data was used for action in their community/at their site

ISCCCE
IMPLEMENTATION SCIENCE CENTER FOR CANCER CONTROL EQUITY

7

End presentation with clear next steps – include who will take each action and by when
Provide a clear ask to the CHC co-authors

Questions?

ISCCCE
IMPLEMENTATION SCIENCE CENTER FOR CANCER CONTROL EQUITY

8

Make sure to save 5 minutes for any final questions
